# Giant Cell Arteritis in the Third Decade of Life: A Case for Expanded Clinical Suspicion

**DOI:** 10.7759/cureus.79262

**Published:** 2025-02-18

**Authors:** Ogheneakpobor E Ubogun, Aishat T Alonge, Chinazo J Okenwa, Ifeoma L Ndigwe, Peace D Akhimienmhona

**Affiliations:** 1 Internal Medicine, Delta State University Teaching Hospital, Oghara, NGA; 2 Internal Medicine, College of Medicine, University of Ibadan, Ibadan, NGA; 3 Internal Medicine, Windsor University School of Medicine, Lagos, NGA; 4 Internal Medicine, Chukwuemeka Odumegwu Ojukwu University Teaching Hospital, Awka, NGA; 5 Internal Medicine, V. N. Karazin Kharkiv National University, Kharkiv, UKR

**Keywords:** corticosteroids, gca, migraine, third decade, visual loss

## Abstract

Giant cell arteritis (GCA), also known as temporal arteritis, is the most common idiopathic systemic vasculitis in middle-aged adults, predominantly involving large and medium-sized arteries. We report an unusual case of a 34-year-old woman with a five-year history of migraines who developed symptoms, including headache, transient vision loss, jaw claudication, and a 5 kg weight loss over two months. Physical examination revealed scalp tenderness, with no other notable findings. Laboratory investigations showed an elevated erythrocyte sedimentation rate (ESR) of 85 mm/hr and a C-reactive protein (CRP) level of 50 mg/L. Ultrasound of the temporal artery demonstrated thickening with the characteristic "halo" sign, and a biopsy confirmed inflammatory changes with multinucleated giant cells. Treatment with high-dose prednisolone resulted in remission, and the patient was discharged with a follow-up plan in place. GCA predominantly affects the superficial temporal arteries, with irreversible vision loss being its most serious complication. Prompt diagnosis and treatment are critical. The diagnosis relies on clinical features, elevated inflammatory markers, imaging studies, and histopathological confirmation. According to the European Alliance of Associations for Rheumatology (EULAR) guidelines, temporal artery ultrasound showing a non-compressible "halo" sign is recommended for diagnosis. Further studies are needed to investigate the potential connection between migraines and GCA. This case underscores the rarity of GCA in individuals under 50 and highlights the possibility of GCA developing in younger patients with a longstanding history of migraines.

## Introduction

Giant cell arteritis (GCA), also referred to as temporal arteritis, is a non-necrotizing granulomatous vasculitis that predominantly affects large and medium-sized arteries [1,2]. It is described as a multifocal, segmental panarteritis [3] and is considered a rheumatological emergency due to its potential to cause rapid, irreversible vision loss and stroke [1]. GCA primarily affects individuals over 50 years of age, with a female-to-male ratio of 3:1. It is more prevalent in Northern European populations, with an incidence of 20-30 cases per 100,000 individuals [1], but is rare in the Caribbean and among Black populations [4,5].

GCA manifests in cranial forms, which involve branches of the carotid, vertebral, and temporal arteries, as well as extracranial forms, often affecting the aorta and its major branches [1,4]. Approximately half of all GCA patients exhibit some degree of aortic involvement [5], and disease relapse occurs in about 80% of cases [1].

The most common symptoms of GCA include headaches, jaw claudication, and ocular manifestations [1]. Up to 50% of patients present with systemic symptoms, such as low-grade fever, malaise, fatigue, anemia, night sweats, anorexia, and weight loss [2,4]. These systemic symptoms are present in 15% of patients at the time of initial diagnosis and in 20% at relapse [4]. Ocular involvement, a hallmark of GCA, is a significant concern due to its potential to cause irreversible vision loss, underscoring the importance of early diagnosis and treatment [2]. Additional symptoms may include scalp tenderness, claudication of the tongue or limbs, peripheral neuropathy, dry cough, sore throat, and polymyalgia rheumatica (PMR), which can precede, coexist with, or follow a GCA diagnosis [2].

This report presents a rare case of GCA in a 34-year-old Nigerian woman and explores the potential link between migraines and GCA, highlighting its implications for clinical practice.

## Case presentation

A 34-year-old Nigerian female high school teacher presented with sudden, transient blindness in her right eye, which resolved spontaneously within 30 minutes. This episode occurred twice within the past week. Along with this, she reported persistent headaches, fatigue, a 5 kg weight loss, and jaw claudication over the past two months. The headache was localized to the temporal region, and it was not amenable to over-the-counter analgesics. She also experienced difficulty chewing and scalp pain.

Her medical history included migraines, diagnosed five years ago. She was taking sumatriptan for migraines and ibuprofen for pain. Family history was significant for hypertension in both parents. She is a lifetime non-smoker but takes alcohol occasionally. The review of systems was otherwise unremarkable.

On examination, the patient was alert with stable vital signs. There was tenderness over the right temporal artery and temporalis muscles, along with scalp tenderness. Visual acuity and the funduscopic exam were normal. Other systems were normal.

Laboratory tests revealed a normal complete blood count, but an elevated erythrocyte sedimentation rate (ESR) of 85 mm/hr and C-reactive protein (CRP) of 50 mg/L (Table 1). Liver, renal, and thyroid function tests were normal. Imaging included a temporal artery Doppler ultrasound scan (TADUS), which showed bilateral temporal artery thickening with a "halo" sign, and a normal brain magnetic resonance imaging (MRI) scan. A temporal artery biopsy (TAB) confirmed GCA, with inflammatory infiltrates and multinucleated giant cells.

**Table 1:** Laboratory investigations

Test Parameter	Result	Normal Range
Complete Blood Count		
Hemoglobin	13.5 g/dL	12-16 g/dL
Hematocrit	40%	36-46%
White Blood Cell Count	6.0 x 10^9/L	4.0-11.0 x 10^9/L
Platelet Count	250 x 10	150-400 x 10
Mean Corpuscular Volume (MCV)	85 fL	80-100 fL
Mean Corpuscular Hemoglobin (MCH)	29 pg	27-32 pg
Erythrocyte Sedimentation Rate (ESR)	85 mm/hr	<20 mm/hr
C-Reactive Protein (CRP)	50 mm/L	<10 mg/L
Renal Function Test		
Blood Urea Nitrogen	15 mg/dL	7-20 mg/dL
Creatinine	0.9 mg/dL	0.6-1.2 mg/dL
Glomerular Filtration Rate (GFR)	95 mL/min	>90 mL/min
Sodium	140 mmol/L	135-145 mmol/L
Potassium	4.2 mmol/L	3.5-6.0 mmol/L
Liver Function Test		
Alanine Aminotransferase (ALT)	25 U/L	7-56 U/L
Aspartate Aminotransferase (AST)	20 U/L	10-40 U/L
Alkaline Phosphatase (ALP)	60 U/L	44-147 U/L
Total Bilirubin	0.8 mg/dL	0.1-1.2 mg/dL
Albumin	4.0 g/dL	3.5-5.0 g/dL
Total Protein	7.0 g/dL	6.0-8.3 g/dL

The patient was started on high-dose corticosteroids (prednisone 60 mg daily) to reduce inflammation and prevent vision loss, along with omeprazole 20 mg daily to prevent gastric ulcers. Weekly ESR and CRP monitoring guided steroid adjustments, and ophthalmologic exams were scheduled to monitor visual complications.

Over the first week, she reported significant improvement in headache severity and resolution of jaw claudication. No further episodes of transient blindness occurred. ESR and CRP decreased to 40 mm/hr and 20 mg/L, respectively. By one month, her energy levels improved, and she experienced no further headaches or jaw pain. ESR and CRP continued to decline to 20 mm/hr and 5 mg/L, leading to a reduction in prednisone to 40 mg daily. Visual exams remained normal.

By three months, the patient was symptom-free, with no recurrence of visual disturbances or jaw claudication. ESR and CRP normalized to 10 mm/hr and <3 mg/L, and prednisone was further reduced to 20 mg daily. By six months, she had returned to her full teaching schedule, with ESR and CRP within normal limits and prednisone reduced to 10 mg daily.

**Figure 1:**
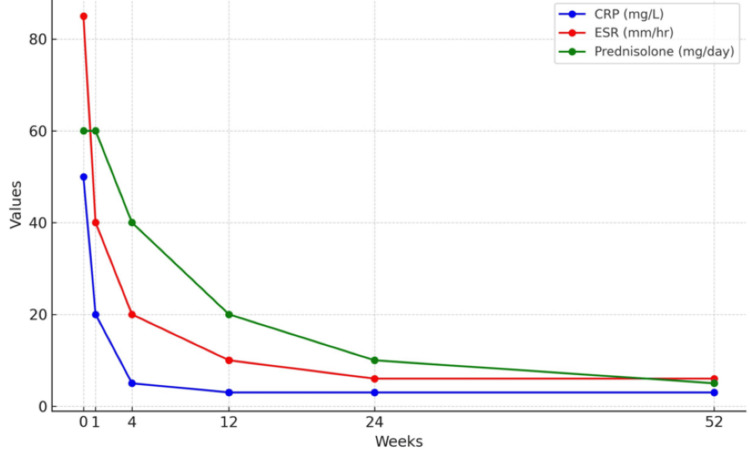
Patient's inflammatory markers monitoring and prednisolone adjustment CRP, C-reactive protein; ESR, Erythrocyte sedimentation rate

At 12 months, the patient remained asymptomatic, with normal inflammatory markers (ESR: 6 mm/hr, CRP: <3 mg/L). Prednisone was reduced to 5 mg daily, and by 24 months, she was in remission, with prednisone discontinued after tapering. She remained on low-dose aspirin for prophylaxis. Figure 1 summarizes the patient's clinical course as it relates to prednisolone dosing and inflammatory markers. Subsequent ophthalmologic investigations, following inflammatory marker monitoring, were unremarkable.

## Discussion

The precise etiology of GCA remains unclear, but it is believed to be triggered by an unidentified event [4]. A theory proposes that infectious agents may act as the initiating factor, particularly in older individuals who are more susceptible to infections and less likely to have primary autoimmune diseases [6]. Potential infectious triggers include varicella-zoster virus, herpes simplex virus, Epstein-Barr virus, parvovirus B19, *Chlamydia pneumoniae*, and *Mycoplasma pneumoniae* [6]. Established risk factors for GCA include advanced age, female gender, the HLA-DRB1*04 allele - common among Northern European populations - high diastolic blood pressure, and smoking [6,7].

This report of a 34-year-old woman with a five-year history of migraines who later developed GCA raises important questions about the potential connection between chronic migraines and systemic inflammatory vasculitis. Migraines, traditionally classified as a neurovascular disorder, may involve prolonged vascular inflammation, potentially priming the immune system for an exaggerated inflammatory response characteristic of GCA [8]. Alternatively, shared genetic or environmental factors may contribute to the development of both conditions [6-8]. This case bears similarity to a presentation reported by Devi et al., who documented GCA in a 61-year-old male with a comparable clinical course [9].

Diagnosing GCA can be complex, especially given the need to distinguish it from several other conditions, including Takayasu's arteritis, polyarteritis nodosa (PAN), syphilitic aortitis, systemic lupus erythematosus (SLE), and idiopathic intracranial hypertension (IIH). It relies on a detailed clinical history, physical examination, and appropriate investigations. Clinical acumen, paired with diagnostic tests, provides the greatest diagnostic sensitivity and specificity [4]. The American College of Rheumatology (ACR) classification criteria (1990) require three out of five criteria for a GCA diagnosis: onset at age ≥50 years, a new localized headache, temporal artery tenderness or reduced pulse, elevated ESR (≥50 mm/hr), and a positive TAB [4,10]. Elevated acute-phase reactants such as ESR, CRP, and platelets are also commonly observed [2]. TAB remains the diagnostic gold standard, showing histologic features such as inflammatory infiltrates with multinucleated giant cells and disruption of the internal elastic lamina, correlating with ischemic symptoms [2].

Recent guidelines from the European Alliance of Associations for Rheumatology (EULAR) recommend TADUS as an alternative first-line diagnostic tool when performed by experienced specialists [11]. TADUS, a non-invasive and accessible method, can detect the "halo" sign, indicative of arterial wall edema and concentric vessel wall thickening [2,12].

The patient’s long-standing migraine history posed a diagnostic challenge, as her new-onset headache could easily have been attributed to her primary condition. However, the emergence of systemic symptoms, including transient monocular blindness, scalp tenderness, jaw claudication, weight loss, and fatigue, alongside abnormal laboratory results (ESR of 85 mm/hr and CRP of 50 mg/L), prompted further evaluation. Diagnostic imaging via TADUS and histopathologic confirmation through TAB ultimately established the GCA diagnosis.

Both the EULAR and the British Society for Rheumatology (BSR) guidelines recommend initiating GCA treatment with high-dose glucocorticoids [11,13]. While effective in inducing remission, relapses are common during dose tapering. This patient was treated with high-dose prednisone (60 mg daily) and monitored closely with ESR, CRP, and ophthalmologic evaluations, achieving remission. Tocilizumab, a novel humanized monoclonal antibody, has been shown to yield significant efficacy in attaining sustained corticosteroid-independent remission. Further, promising treatment results have also been shown with azathioprine, leflunomide, and mycophenolate mofetil [14,15]. These medications were not used in our patient due to cost and the fact that prednisolone was sufficient. This case underscores the importance of maintaining a high index of suspicion for GCA in younger patients presenting with atypical headaches, even in the context of a known migraine history.

## Conclusions

This case underscores the possibility of GCA developing in younger patients with a long-standing history of migraines. It highlights the importance of maintaining high clinical vigilance when evaluating secondary causes of headaches, as imminent harm may abound. Further research is needed to investigate the pathophysiological relationship between these conditions and refine diagnostic approaches.
